# Structural basis of the complementary activity of two ketosynthases in aryl polyene biosynthesis

**DOI:** 10.1038/s41598-021-95890-y

**Published:** 2021-08-11

**Authors:** Woo Cheol Lee, Sungjae Choi, Ahjin Jang, Jiwon Yeon, Eunha Hwang, Yangmee Kim

**Affiliations:** 1 Department of Bioscience and Biotechnology, Konkuk University, Seoul, 05029 South Korea; 2 Bio-Chemical Analysis Team, Korea Basic Science Institute, Ochang, 28119 Chungcheongbuk-do South Korea

**Keywords:** X-ray crystallography, Enzyme mechanisms

## Abstract

Aryl polyenes (APE) are one of the most widespread secondary metabolites among gram-negative bacteria. In *Acinetobacter baumannii*, strains belonging to the virulent global clone 2 (GC2) mostly contain APE biosynthesis genes; its relevance in elevated pathogenicity is of great interest. APE biosynthesis gene clusters harbor two ketosynthases (KSs): the heterodimeric KS-chain length factor complex, ApeO-ApeC, and the homodimeric ketoacyl-acyl carrier protein synthase I (FabB)-like KS, ApeR. The role of the two KSs in APE biosynthesis is unclear. We determined the crystal structures of the two KSs from a pathogenic *A. baumannii* strain. ApeO-ApeC and ApeR have similar cavity volumes; however, ApeR has a narrow cavity near the entrance. In vitro assay based on the absorption characteristics of polyene species indicated the generation of fully elongated polyene with only ApeO-ApeC, probably because of the funnel shaped active site cavity. However, adding ApeR to the reaction increases the throughput of APE biosynthesis. Mutagenesis at Tyr135 in the active site cavity of ApeR reduces the activity significantly, which suggests that the stacking of the aryl group between Tyr135 and Phe202 is important for substrate recognition. Therefore, the two KSs function complementarily in the generation of APE to enhance its production.

## Introduction

Fatty acid synthases (FASs) participate in essential cellular processes required for the generation of the lipid membrane bilayer. Polyketide synthases (PKS) are evolutionarily related to FASs and they generate numerous secondary metabolites^1–3^. In FAS, β-ketoacyl-acyl carrier protein (ACP) synthase (KAS, FabB, or FabF in *Escherichia coli*) is responsible for the chain elongation using a two-carbon unit from malonyl-ACP through decarboxylative Claisen condensation^4^. The subsequent action of β-ketoacyl ACP reductase (FabG) and β-hydroxyacyl ACP dehydratase (FabZ) leads to the formation of β-enoyl ACP. Each elongation step terminates with the action of β-enoyl reductase (FabI) for generating fully oxidized linear alkyl chains. There are two major types of FAS, based on the scheme of protein association during the concerted iteration of chain elongation. Type I FASs are megasynthases containing multiple protein modules in a single open reading frame. Type II FASs are mostly of bacterial origin and they catalyze fatty acid synthesis by shuttling acyl chains from ACPs to FAS proteins, which are expressed independently^5,6^.

PKS synthesizes diverse natural products through a chemical process similar to that of FAS and the synchronous functioning of ketosynthase (KS), ketoreductase (KR), dehydratase (DH), and enoylreductase (ER) encoded in the megasynthases (type I PKS) or in the smaller iterative modules (type II PKS). Based on the degree of reduction on keto groups, PKSs are grouped into non-reducing (NR), partially reducing, and highly reducing (HR) PKSs. In case of NR-PKSs, the limited action of β-KR during the elongation steps leads to highly reactive polyketide intermediates, which readily cyclize to form aromatic compounds. The mechanism of HR-PKS is more similar to that of FAS; the final step of ER is skipped often to generate compounds with conjugated double bonds. These polyene polyketides, produced by various actinomycetes and fungi, display diverse bioactivities^7^.

There is a large family of biosynthetic gene cluster (BGC) coding for aryl polyene (APE) biosynthesis genes^8^. These BGCs are common in gram-negative bacteria and the enzymes catalyze the synthesis of 4-hydroxybenzoyl (4HB) polyene compounds with 6 to 7 double bonds. The precise role of APE species is still unclear; however, the frequent presence of APE BGC in symbiotic and pathogenic bacteria, leads to the speculation that host immune system evasion is its primary function. Recently, the proteins encoded by APE BGC from *Xenorhabdus doucetiae*, a symbiotic bacterium, have been studied extensively^9^. A KS-chain length factor (CLF) heterodimer (ApeO-ApeC) possibly functions as the condensation enzyme, and other modification enzymes such as ApeQ (KR) and ApeH-ApeP (DH) were identified and characterized^9,10^. We found that *Acinetobacter baumannii*, a notorious nosocomial pathogen, contains APE BGC, especially among the global clone 2 (GC2), which have enhanced virulence^10^.

**Figure 1:**
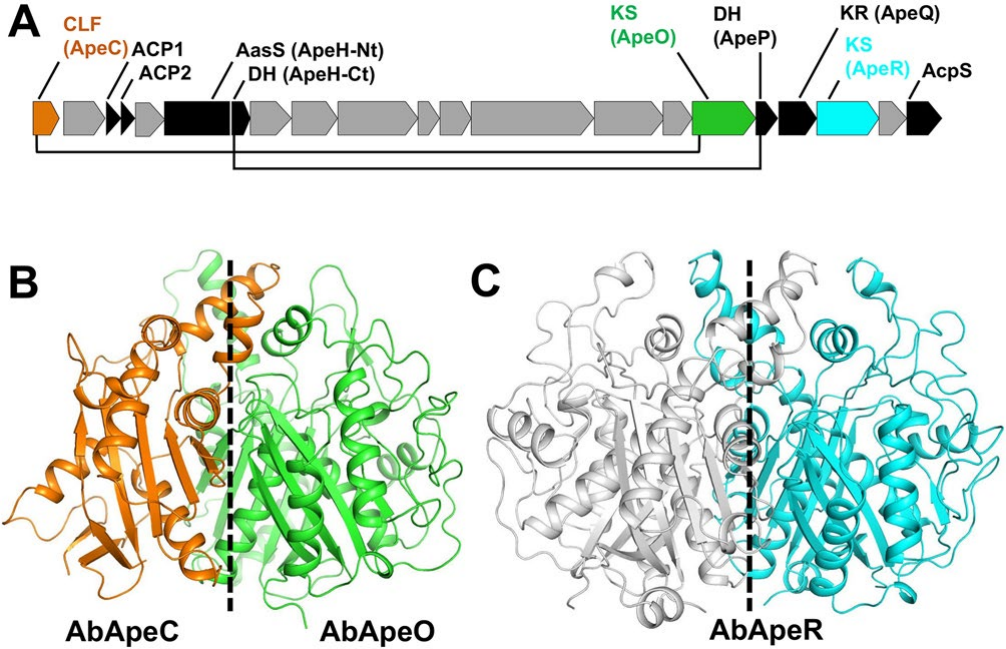
(**A**) APE biosynthesis gene cluster showing the location of the two ketosynthases (KSs) ApeO and ApeR. The two KSs, AbApeO and AbApeR, are indicated in green and cyan, respectively. AbApeC is in orange. Proteins with function associated with FAS or PKS are in black. Protein coding genes with unknown function are in gray. The line linking two genes indicates association of the proteins in their quaternary structures. (**B**) Overall cartoon representation for AbApeO-AbApeC. The two-fold (pseudo) rotational axis of the dimeric enzymes is indicated using dashed lines. (**C**) Overall ribbon model of AbApeR. The two-fold rotational axis of the dimeric enzymes is indicated using dashed lines.

One of the characteristics of APE BGC is two independently translated ACPs, ACP1 (ApeE), and ACP2 (ApeF), which are present consecutively within the cluster (Fig. 1A). Multiple ACP domains in FAS or PKS are often found in a beads-on-a-string configuration for coordinating complex editing of substrate in polyunsaturated fatty acid biosynthesis^11,12^; however, it is unusual to find free ACPs working in concert. The precise role of each ACP is still unclear; however, it is presumed that ACP1 provides the starter molecule, 4HB-ACP1, whereas ACP2 is required for the delivery of malonyl groups to the growing polyene chain^9^. Distinct interaction patterns between ACPs and KS-CLF in APE BGC are expected. Another feature of APE BGC is the existence of a second KS in the APE gene cluster (Fig. 1A). The gene coding for this protein, ApeR, is located between the genes for ApeQ and a protein with unknown function. ApeR also functions as a KS with APE biosynthesis proteins in vitro. However, the products from ApeR are mostly shorter than those from ApeO, generating polyene species with up to four double bonds^9^. The cavity volume and hydrophobicity in a KS is correlated with the size of the product and affects the overall throughput of the PKS^13,14^. Therefore, evaluating the number of iterations performed by KS^14^ and determining the high-resolution structure of ApeR is critical in understanding the function of this redundant KS.

In PKS, a CLF is necessary for the chain length control, which is determined by the pocket depth^15,16^. Several structures of KS-CLF complexes are available, such as that of ishigamide biosynthetic KS-CLF (Iga11-Iga12) in complex with cognate ACP^17^. Ishigamide is a secondary metabolite containing four conjugated bonds with an unknown function; it is isolated from the genera, *Actinomycetes*^18^. In the complex structure, ACP interacts with CLF mainly at helix II. In the active site, a charged residue, Asp113, promotes the release of β-ketoacyl ACP by electrostatic repulsion for further modification by Iga13 (KR) and Iga16 (DH). The structure of KS-CLF (AntD-AntE) from a minimal PKS system for the biosynthesis of anthraquinone was elucidated^19^; contrary to that in Iga11-Iga12, the active site cavity allows the stabilization of polyketide species, and the octaketide-ACP intermediate is released after the seventh round of condensation, for further modification. In a highly reducing polyketide APE, it is expected that the APE-ACP intermediate is readily released to be modified by ApeQ and ApeH-ApeP.

To understand the structural basis of the functional redundancy in the two KSs, AbApeO-AbApeC and AbApeR, from the APE BGC of *A. baumannii*, we determined the crystal structures of AbApeO-AbApeC and AbApeR in the absence of substrates. The crystal structure of ApeO-ApeC from *X. doucetiae* is known^9^, but the structure of ApeR has not been published, to the best of our knowledge. The relationship between FAS and PKS is a fascinating subject in terms of bacterial evolution and systems biology. The APE gene cluster consists of two KSs with characteristic features of PKS (ie ApeO-ApeC) and FAS (ApeR) is intriguing. The structure–function relationship assisted by high resolution structures is necessary to study the role of each KS. Furthermore, detailed structural analyses of the two KSs are invaluable in probing the protein–protein interaction between two cognate ACPs, ACP1 and ACP2, another unique feature of APE BGC. Our depiction of the joint structures of two KSs in APE BGC will shed light on APE in the synapse of the microbe–host interface.

## Results

### The crystal structures of AbApeO-AbApeC and AbApeR

**Figure 2:**
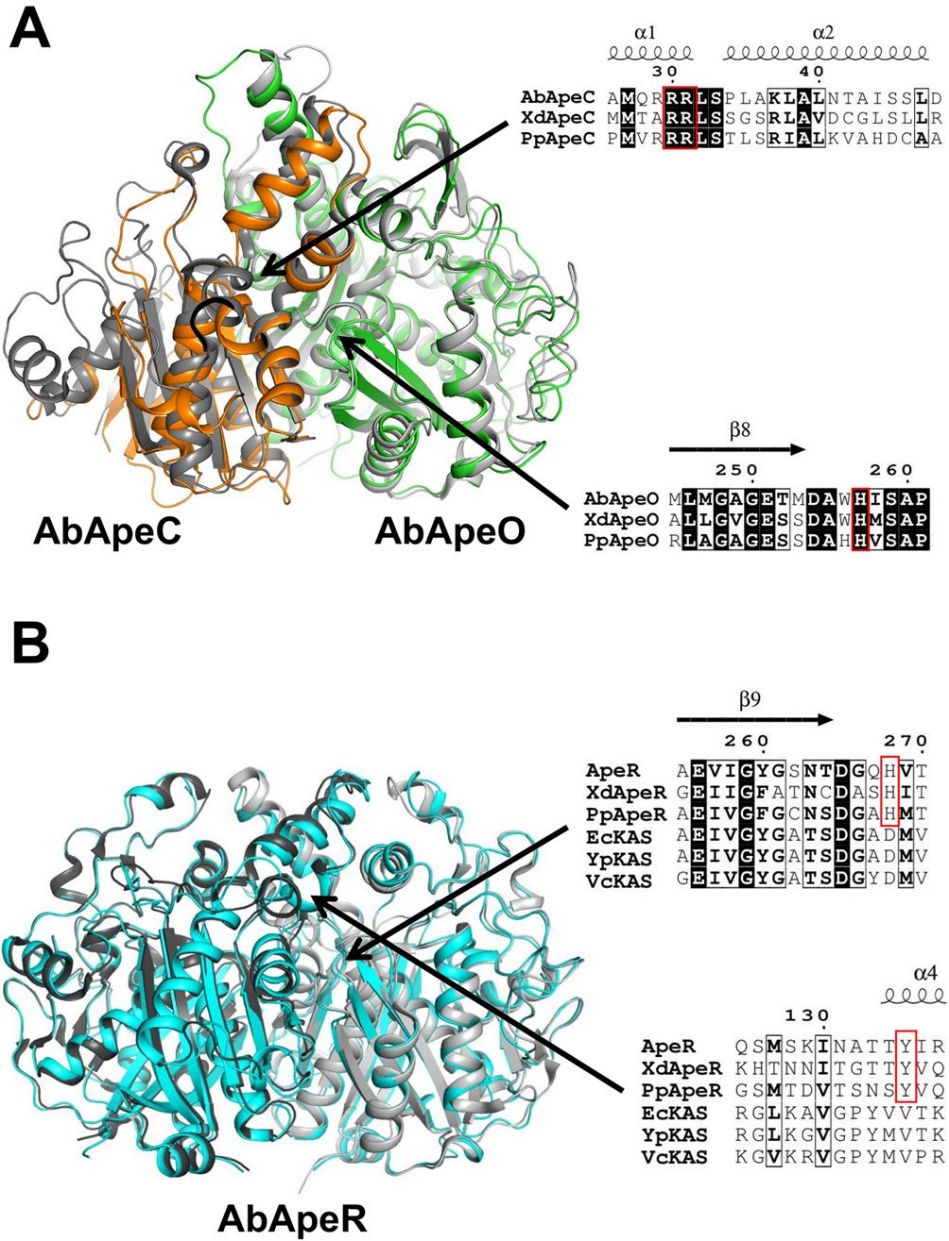
The overall structures of AbApeO-AbApeC and AbApeR. (**A**) AbApeO-AbApeC and (**B**) AbApeR with each protomer are indicated using cyan or green color. Each protein was superposed with XdApeO-XdApeC (**A**) or a protein with unknown function (**B**; PDB ID 4EWG, *Paraburkholderia phymatum* STM815 or PpApeR) with one protomer (dark gray color). EcFabB, *Escherichia coli* K12 FabB; YpFabB, *Yersinia pestis* FabB; VcFabB, *Vibrio cholerae* O1 biovar El Tor str. N16961 FabB.

The structure of AbApeO-AbApeC heterodimer was determined by molecular replacement using the coordinates of ApeO-ApeC from *X. doucetiae* (PDB ID: 6QSP), which shares 45% and 33% amino acid sequence identity with AbApeO and AbApeC, respectively. The crystals belong to the space group of *P*2_1_ with cell dimensions of *a* = 54.71, *b* = 104.24, *c* = 98.18 Å, and *β* = 98.2°. As previously observed in 6QSP, AbApeO-AbApeC complex forms a heterodimer with pseudo two-fold rotational symmetry (Fig. 1B)^9^. In the crystal form, there were two heterodimers in the asymmetric units; we focused on the structure of the first heterodimer (chain A and chain B), which had a relatively lower overall temperature *B*-factor than that of chain C and chain D in the structure. The overall structure was superposed with the *X. doucetiae* KS-CLF complex with an r.m.s.d. of 0.68 Å (Fig. 2A). A noticeable difference occurred at the N-terminus; around 20 residues were truncated in AbApeC. The truncated residues formed the first helix in XdApeC and *Paraburkholderia phymatum* ApeC (PpApeC) (Fig. 2A, arrow). We compared the structures of XdApeC/O and AbApeC/O. In APE KS-CLF heterodimer, CLF is about half the size of the KS counterpart. KS is normally composed of two duplicate thiolase folds^20^. However, AbApeC was composed of only one thiolase fold domain. Structurally, the first thiolase domain was mostly retained, whereas the second thiolase domain was almost lost, except for few β-strands.

The crystal structure of AbApeR was solved by molecular replacement using the coordinates of a protein with unknown function sharing ~ 59% identity (PDB ID: 4EWG). The crystal structure contained four molecules of AbApeR in the asymmetric unit. AbApeR was a homodimeric protein with twofold symmetry (Fig. 1C), and there were two homodimers in the crystal structure. We focused on the structure of chain A and chain B, the homodimer with the low B-factors. The space group was *P*2_1_ with cell dimensions of *a* = 62.58, *b* = 109.53, *c* = 122.90, and *β* = 97.4º. AbApeR and *E. coli* KAS I protein (EcFabB) were remarkably similar (Fig. 2B). The proteins shared an amino acid sequence identity of 39% and the proteins could be superposed with an r. m. s. d. of 0.886 Å. There is a protein structure in PDB (4EWG), which shares ~ 59% identity with that of AbApeR. The two structures could be superposed with an r.m.s.d. of 0.574 Å. This ApeR homolog is isolated from *Paraburkholderia phymatum* STM815 (PpApeR) and annotated as a β-ketoacyl synthase. However, evaluation of the gene cluster containing this protein strongly suggests it to be an ApeR protein.

### Conserved residues on the surface of the two KSs

**Figure 3:**
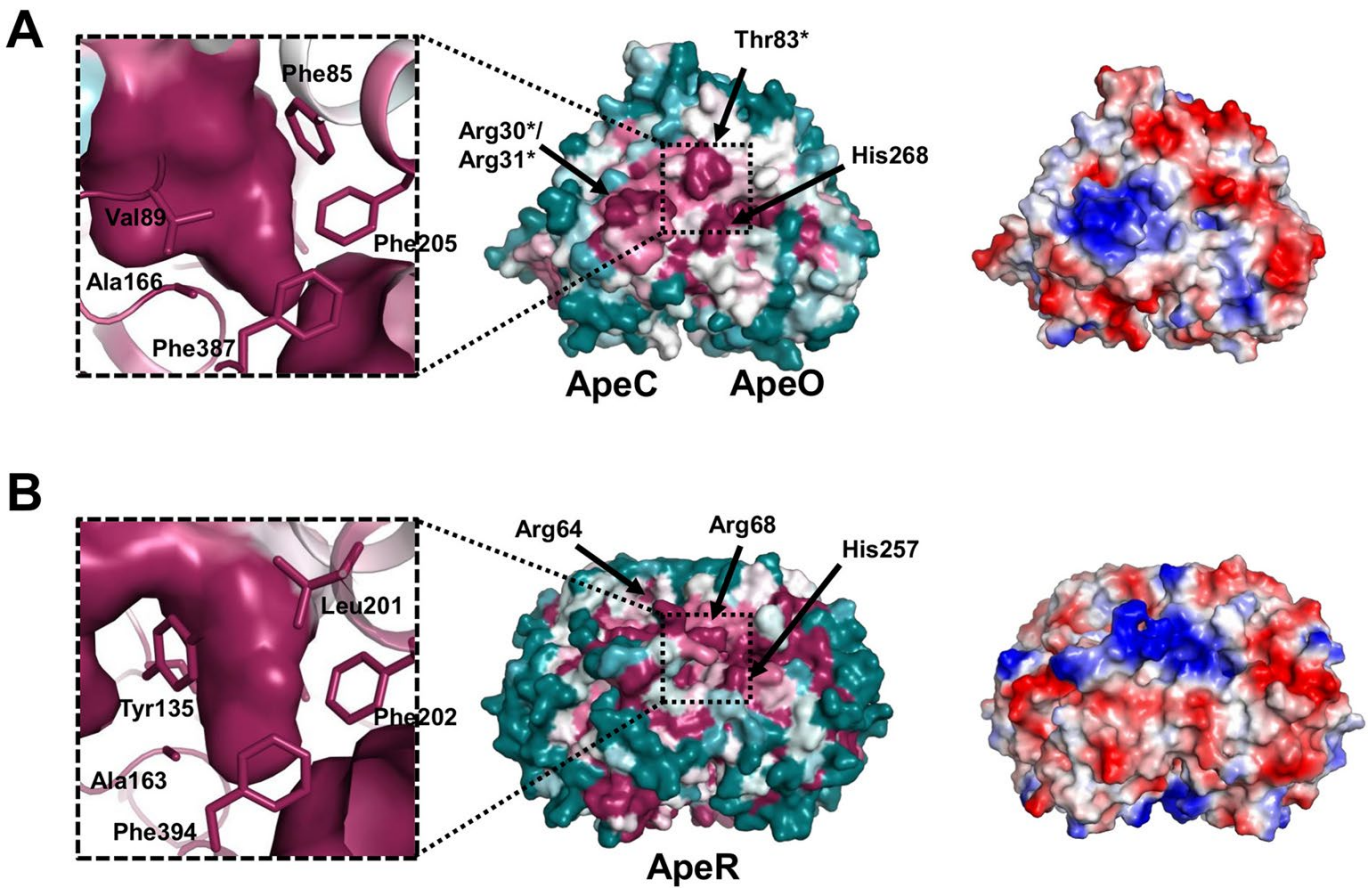
The surface representation of AbApeO-AbApeC and AbApeR. (**A**) AbApeO-AbApeC and (**B**) AbApeR. ConSurf and surface electrostatic potential renderings are displayed in the middle, with the inset on the left showing a magnified view of the cavity. Surface electrostatic potential image of each protein complex is displayed in the right panel in the same orientation as that in the ConSurf rendering.

To understand the protein–protein interaction between ACP1 or ACP2 with AbApeO-AbApeC heterodimer or AbApeR homodimer, we analyzed the conservation of the surface residues using ConSurf server with default parameters^21^. On the surface, AbApeC had Arg30, Arg31, and Thr83; AbApeO has His257 residues. These residues were conserved among the various APE biosynthesis KS-CLF homologs (Fig. 3A). Among the conserved residues, Arg30 and Arg31 residues in AbApeC appeared to be important for the ACP-KS-CLF interactions, because ACPs are negatively charged proteins. The residues were present on the C-terminal side of helix 1 in AbApeC (see Supplementary Fig. S1 online) and they were aptly located for the interaction with incoming ACPs. His268 residue in AbApeR was a part of the loop sterically linked to the loop containing the gating residue Phe391 in *E. coli* FabB (see Supplementary Fig. S2 online)^22^. Inside the AbApeO cavity, in addition to the gating residue Phe387, residues Phe85, Val89, Ala166, and Phe205 near the entrance of the active site cavity were strictly conserved, underlining the importance of these residues in function (inset of Fig. 3A).

Arg64 and Arg68 were conserved among the surface residues in AbApeR. The location of His268 in ApeR was similar to that of His257 in AbApeO-AbApeC (Fig. 3B and see Supplementary Fig. S2 online), and it appeared to be important in APE biosynthesis. Inside the cavity, Tyr135, Ala163, Leu201, and Phe202 residues were lined near the entrance regulated by the gating residue Phe394 (Fig. 3B). The cavity volume of AbApeR near the entrance is reduced, when compared to that of AbApeO-AbApeC, because of the presence of Tyr135.

### Residues lining the cavity

**Figure 4:**
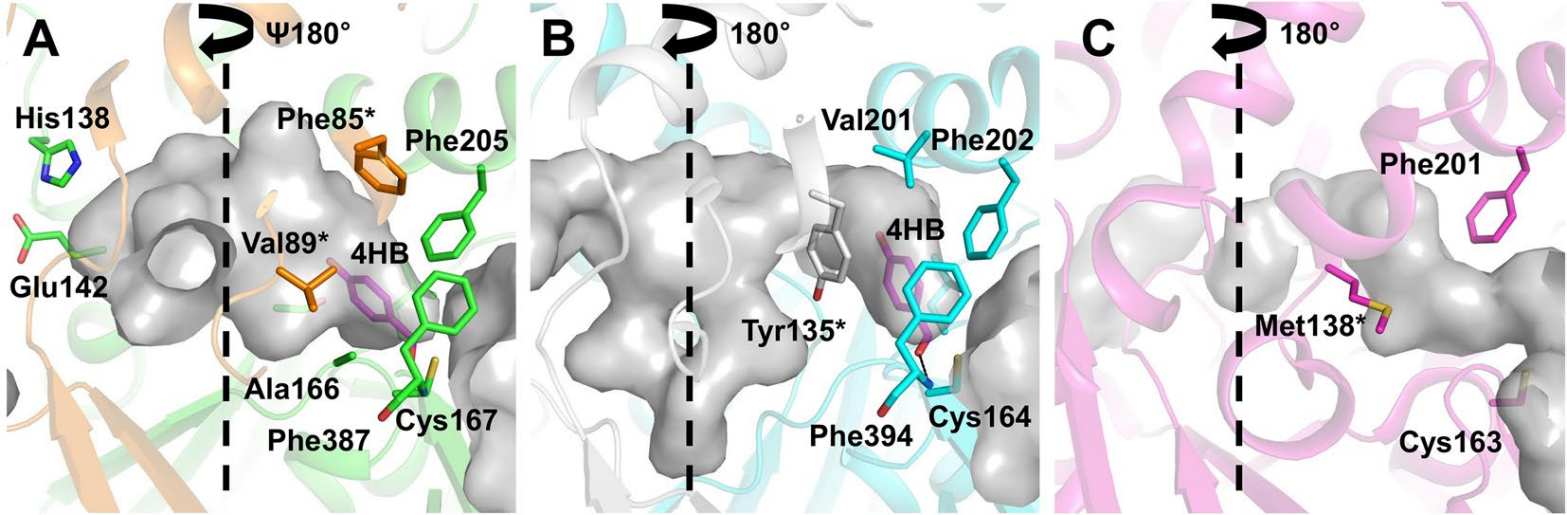
Comparison of cavity shape, volume, and lining residues. (**A**) AbApeO-AbApeC, (**B**) AbApeR, and (**C**) EcFabG. The two-fold rotational axis of the dimeric proteins is indicated using dashed lines. Residues from AbApeC or homodimeric partner (AbApeR or EcFabG) are indicated using asterisks. 4HB is rendered in magenta.

The cavity of AbApeC-AbApeO was characterized by its asymmetric shape because of the lack of cavity belonging to AbApeC as observed in other CLFs (Fig. 4A). The cavity was lined by residues from both AbApeO and AbApeC (Fig. 4A). The volume of the cavity was 120.4 Å^3^, as calculated using the SiteMap tool of Maestro software package, 2020–2 release. To understand the implications of the AbApeO-AbApeC structure, we compared it with that of AntDE or Iga11-Iga12 complexes. Each of these proteins could be superposed with AbApeO-AbApeC with an r.m.s.d. of 1.314 or 0.910 Å, respectively. In ishigamide synthase, the residues lining the cavity are largely hydrophilic including Asp113, which is important for the release of acyl-ACP intermediate for reduction and dehydration; however, we could not find a similar residue in the superposed structure of AbApeO-AbApeC (see Supplementary Fig. S3 online).

The cavity of AbApeR was symmetrical and connected between the homodimeric partners, forming an elongated, narrow canal across the dimer (Fig. 4B). The residues lining the cavity of AbApeR were moderately hydrophobic. The volume of the cavity was 120 Å^3^, which was considerably similar to that of the AbApeO-AbApeC heterodimer. The cavity volume was compared to that of other similar proteins; *E. coli* FabB protein (PDB ID, 2VB9) was 74.4 Å^3^, whereas FabF protein (PDB ID, 1B3N) was 153.3 Å^3^. FabB generates fully oxidized alkyl chains, whereas FabF could produce unsaturated alkyl chains, thus requiring an expanded cavity.

The specific residues lining the cavity were compared to get an insight into the functional evolution of AbApeR. The residues lining AbApeR corresponded to that of Met138 and Phe201 of EcFabB (Fig. 4C). Phe392, the gating residue in FAS KS^22^, was conserved. However, among the residues lining the cavity, Tyr135, Phe202, Leu229, Leu336, Tyr135, and Leu229 were not conserved between EcFabB and AbApeR (see Supplementary Fig. S2 online). In addition to the secondary structural deviation, these residues contributed to the differences in the cavity size and shape between AbApeR and EcFabB.

The KS-CLF in APE biosynthesis requires the benzoyl group to enter the active site. In our previous modeling study with KR (ApeQ) in APE biosynthesis, a conserved leucine residue interacts with the 4HBA moiety^10^. We placed a 4HBA moiety into the active site of AbApeO-AbApeC and AbApeR. The 4-hydroxybenzoate thioester intermediate was created by 4-HB forming a thioester bond with the active site residues or active site nucleophiles Cys167 (AbApeO) or Cys164 (AbApeR). (Fig. 4) In the model of 4HB-AbApeO-AbApeC complex, 4HB moiety was located between Ala166 and Phe205. The cavity shape was oval, ending with two residues, His138 (AbApeO) and Glu142 (AbApeC) (Fig. 4A). The side chains of Tyr135 and Phe205 residues were arranged such that the 4HB moiety was stacked between the two aromatic rings (Fig. 4B).

### Assay to assess polyene biosynthesis by AbApeR and AbApeO-AbApeC

**Figure 5:**
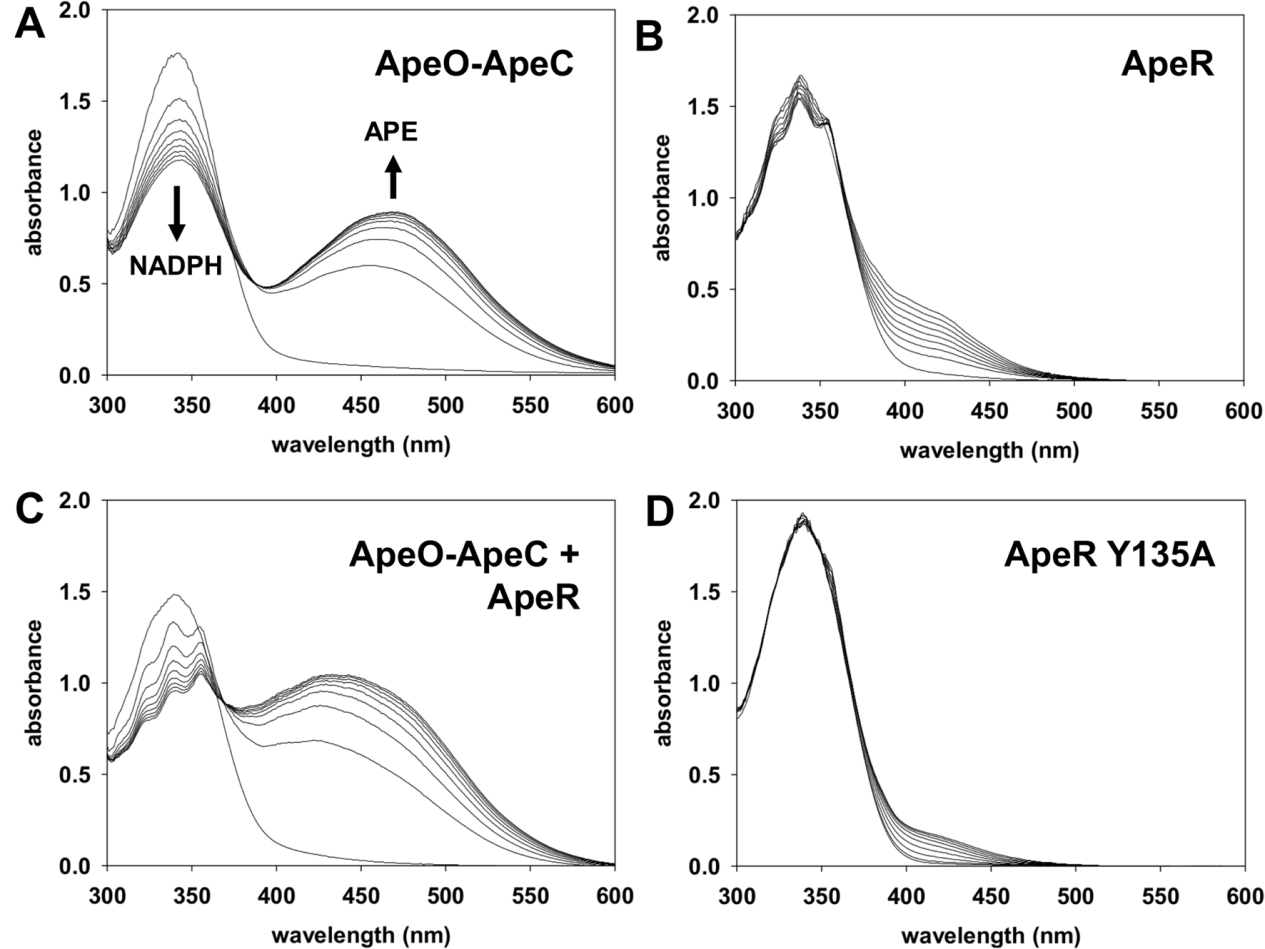
Cumulative spectra recorded during the in vitro spectrophotometric assay of APE generation by AbApeO-AbApeC or AbApeR. Each assay displays a spectrum during the reaction. The spectra are superposed to determine the decrease in NADPH at 340 nm (indicated by the downward arrow) and increase in the seven-membered polyene at 460 nm (upward arrow). Each assay contains 0.125 mM of KSs, except for C, which contains 0.0625 mM each of AbApeO-AbApeC and AbApeR mixed together.

We employed recombinant proteins for APE biosynthesis to assess the roles of AbApeO-AbApeC or AbApeR with ACP1 or ACP2. In AbApeO-AbApeC, a gradual increase in the polyene species with a peak at 462 nm was observed (Fig. 5A); however, only a linear increase in the region spanning 350–500 nm was observed for AbApeR (Fig. 5B). When the two proteins were mixed at equal concentrations, an overall increase in the generation of APE species was observed (Fig. 5C). The overall increase in throughput is more than two fold for AbApeO-AbApeC because only half the amount was used for the assay. To understand the contribution of Tyr135 to the cavity volume and shape, we mutated Tyr135 of AbApeR to alanine and compared the relative activity using a spectrophotometric assay based on the absorption at 430 nm. The activity of the Y135A mutant was reduced by nearly three fold (Fig. 5). Tyr135 was positioned at the beginning of the cavity, where the first 4HB moiety binds. The weak binding with the mutant reduced the activity.

In the spectra of the reaction products produced by AbApeR or AbApeR + AbApeO-AbApeC mixture, there was a peak at 355 nm and a shoulder at 325 nm, separated by approximately 30 nm (Fig. 5B,C). These two absorption wavelengths were presumed to correspond to the peaks for the five conjugated double bonds. However, we could not observe such a spectral feature for the AbApeO-AbApeC reaction products (Fig. 5A).

### Binding of APE KSs to ACP1 or ACP2

**Figure 6:**
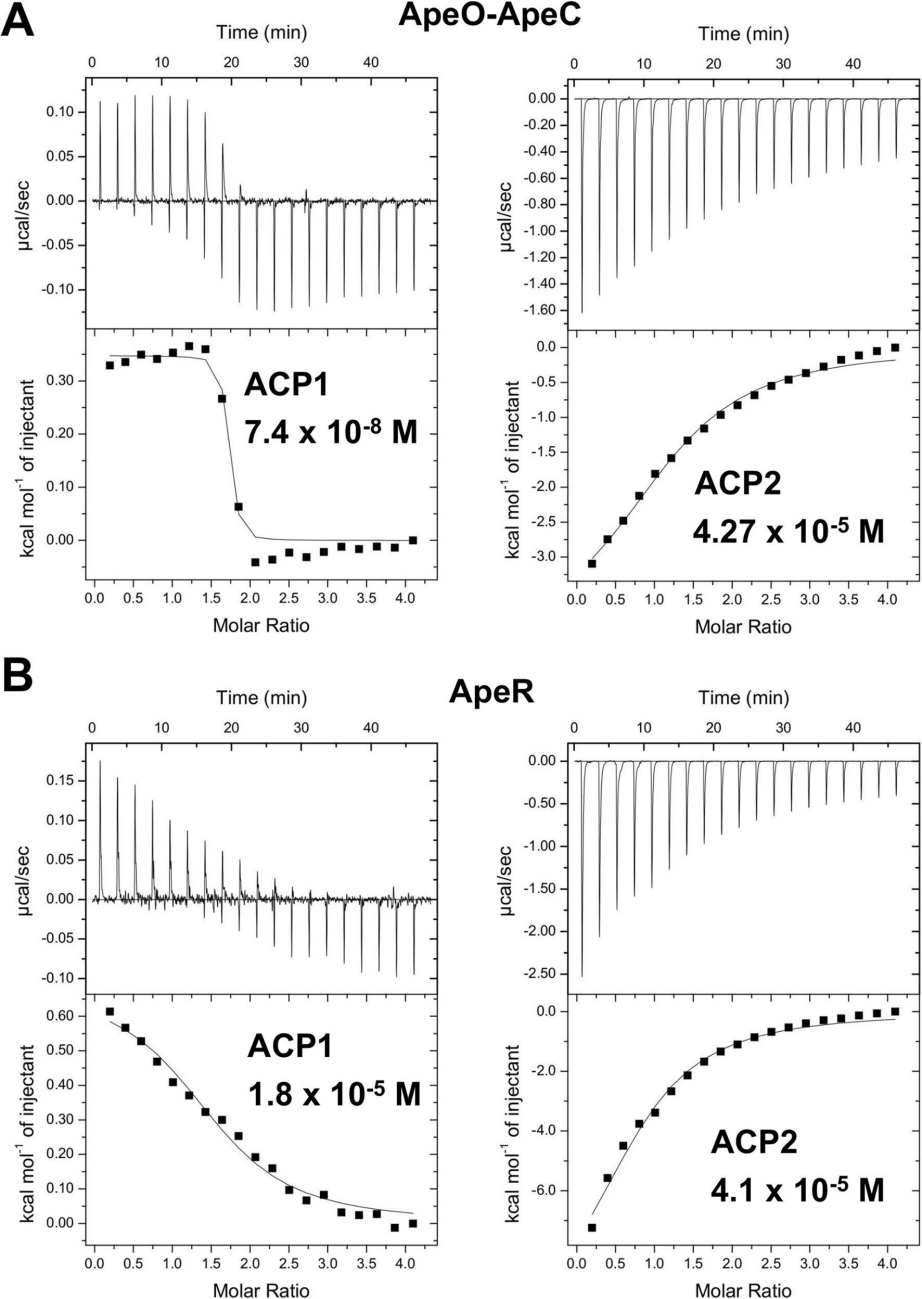
ITC results of AbApeO-AbApeC or AbApeR with holo ACP1 or holo ACP2. The fitted ITC curved graph of (**A**) AbApeO-AbApeC versus holo ACP1 or holo ACP2 and (**B**) AbApeR versus holo ACP1 (sample).

The two ACPs in APE BGC are presumed to have distinctive functions in polyene elongation, and therefore, different affinities for each KS are expected. To measure the binding affinity of two ACPs with ApeO-ApeC or ApeR, ITC analysis was conducted. We employed holo ACPs for the study, so that the 4′-phosphopantetheine (4′-PP) arm could also contribute to the binding. The temperature was set to 25 ºC. Holo ACP1 and holo ACP2 had dissociation constants (*K*d) of 1.8 × 10^−5^ (N = 1.55) and 4.1 × 10^–5^ M (N = 0.71), respectively, for ApeR (Fig. 6A). AbApeO-AbApeC had a comparatively stringent *K*d of 7.4 × 10^−8^ M (N = 1.63) for holo ACP1. However, the *K*d for holo ACP2 was 4.27 × 10^−5^ (N = 1.21), which was comparable to that with AbApeR (Fig. 6B).

The thermogram of each ACP was similar between AbApeR and AbApeO-AbApeC. The profiles of holo ACP2 were exothermic for AbApeR and AbApeO-AbApeC. The profiles of holo ACP1 were transient, in terms of binding isotherm and the transition from endothermic to exothermic states (Fig. 6). The binding was entropy-driven, with the entropy terms (− TΔS) being − 8.2 and − 5.8 (kcal/mol) for AbApeO-AbApeC and AbApeR, respectively.

## Discussion

The structures of two KSs in APE biosynthesis, AbApeO-ApeC and AbApeR, were analyzed to elucidate their functional redundancy in APE biosynthesis. The presence of two KSs in the APE biosynthesis gene cluster is similar to that of iterative PKS, where two KSs exist with orthogonal specificities to ACPs in some aromatic PKS^23^. A KAS III (FabH) like KS was responsible for the priming of the non-acetyl primer unit to its orthogonal ACP. However, APE biosynthesis required AasS-like protein (ApeH-Nt), which was dedicated to the attachment of benzoyl group to ACP1. Therefore, AbApeR and AbApeO-AbApeC have functional roles other than the priming of the benzoyl group. They could act as a completing unit (AbApeO-AbApeC), in contrast to being the rapid but short polymerization unit (ApeR). ApeR was much more efficient in generating polyene species much shorter than that produced by AbApeO-AbApeC, resulting in a characteristic absorption spectrum near the four-double-bond polyene-binding region. This absorption spectrum pattern was almost absent in the reaction with only AbApeO. The generation of short APE species was most pronounced when the two KSs were mixed. This could possibly be attributed to the differences in the affinity of each KS towards ACP1, which promoted the shuttling of ACP1 during the condensation, resulting in an overall increase in the throughput of both the short and long APE species. When the two proteins were mixed, the complementing effect increased the overall throughput of the system by more than two folds. The synergistic effects possibly stem from the different affinities of the two KSs to each tandem ACP protein in the cluster.

**Figure 7:**
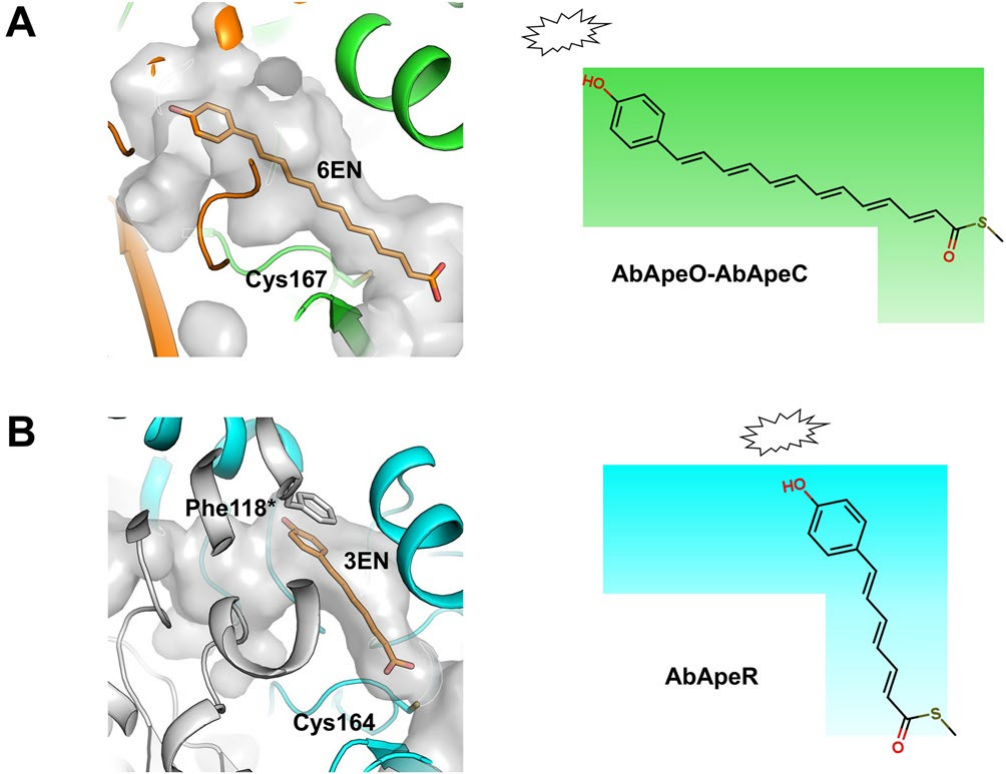
The schematic explanation of differing product lengths based on the shape of cavities for AbApeO-AbApeC (**A**) and AbApeR (**B**). Carboxylate form of aryl polyene with 6-double bonds (6EN) or 3-double bonds (3EN) are docked into the cavity using Glide application of Maestro program package. For each protein, the cavity is rendered in semi-transparent surface with some residues rendered in stick models. On the right panel, the schematic illustration of cavity shape and resultant product length is presented with the steric hindrance as jagged balloons.

The volumes of the active site cavities in AbApeO-AbApeC and AbApeR were very similar; however, the lengths of the products differed. We modeled carboxylate forms of APE with 3 double bonds (3EN) or 6 double bonds (6EN) by the docking software Glide in Maestro software package (Fig. 7). Due to the rigid property of proteins during the docking procedures, 6EN could not fit fully into the cavity relative to the active site Cys167 residue (Fig. 7A). However, it appears 6EN can occupy the cavity with induced fit of the active site residues. For 3EN, the benzyl group is stacked underneath Phe118, a conserved residue among ApeR homologs (Fig. 7B, Fig. S2). In the model of 3EN-AbApeR, the narrow binding site of 3EN moiety led to a dead end with approximately the length of three-double-bond polyene. However, the 6EN-AbApeO-AbApeC model indicated that the bound substrate entered the cavity, such that the full elongation of the substrate up to six conjugated double bonds was possible, as observed previously in XdApeO-XdApeC (Fig. 7)^9^. These models explained the product profile of the two KSs (Fig. 7). In HR-PKS, the growing chains are released right after the condensation. In Iga11-Iga12, the charged Asp113 is involved in product repulsion. However, there was no such residue in the AbApeO-AbApeC and AbApeR structures. We assumed that the limited flexibility of the polyene structure presents conformational restraints in the cavity such that further elongation of polyketide species is disallowed and released after each condensation.

APE containing polyketides alterochromides have a similar, but shorter polyene moiety in their structures than the APE with seven double bonds^23^. The polyene moiety is synthesized by FAS-like proteins and the presence of FAS proteins in these polyketides is analogous to APE biosynthesis. However, alterochromide BGC lacks a KS-CLF enzyme. Considering that short species are readily generated by KAS I-like KSs, similar biosynthesis of short APE chains can only be possible by homodimeric KSs. Both AbApeO-AbApeC and AbApeR did not contain an acidic residue in the active site cavity, such as Asp113 in Iga11, which served as a repulsion notch to release a keto group containing ACPs. Therefore, we suspect a different mode of ACP release for APE biosynthesis modules, in a manner similar to that in FAS enzymes, where steric hindrance at substrate binding cavity prohibits polyketide intermediates to accumulate in the active site pocket^24^. The steric hindrance was considerable for AbApeR, where the 4HB moiety binding Tyr135 and Phe205 impose steric hindrance to further condensation of the bound APE species.

The complementary effect of AbApeR could arise from the structural difference with AbApeO-AbApeC and the different specificities against the two ACPs, especially ACP1. AbApeO-AbApeC exhibited very high affinity towards ACP1, when compared to that by AbApeR. The difference decreased significantly with ACP2. The different affinities between ACP1 and the two KSs could contribute to the generation of shorter products by ApeR, to function as a booster in the early phase of APE elongation. In the ACP-KS interface, a histidine residue (His257 in AbApeO-AbApeC and His268 in AbApeR) was conserved on the surface (Fig. 3). These residues are a part of the loop which is sterically coupled to the gating loop; they appear to interact with an acidic residue from cognate ACP proteins, similar to that in Iga10 (ACP) and Iga12 (CLF) complex structure^7^. Structure–function analysis of ACP1 and ACP2 in solution is currently underway by our research group.

In conclusion, we have determined the structures of two KS proteins, AbApeO-AbApeC and AbApeR, in APE BGC of the virulent strain *A. baumannii* GC clone 2. The two KSs have very different reaction profiles against growing APE chains, as observed in real time using spectrophotometric assays. Combining the two KSs dramatically increased the overall throughput of APE. The functional studies on AbApeO-AbApeC and AbApeR are limited by the lack of cognate ACP structures. However, the structure of two pivotal condensation enzymes of APE biosynthesis indicates structure–function relationship of the products, based on the cavity structures. The structural and functional analyses will enable better understanding of this FAS-PKS hybrid system in the natural products of polyketides and in developing novel antimicrobials.

## Methods

### Cloning, expression, and purification

AbApeO and AbApeC were cloned into the multiple cloning site 1 and 2 of pETDuet-1 expression vector (Novagen, Madison, WI) using *Bam*HI-*Sal*I and *Nde*I-*Xho*I restriction sites, respectively. AbApeO contains a N-terminal His6-tag, which is not cleavable. *E. coli* BL21 (DE3) harboring AbApeO/AbApeC expression vector was cultured at 37 °C and protein expression was induced by adding 0.2 mM IPTG and incubated further for 10 h at 25 °C. The heterodimer was purified using Chelating Sepharose HP 5-ml and Q-Sepharose HP 5-ml columns (GE Healthcare, Chicago, IL) with a gradient of 0 to 1 M NaCl or 0 to 500 mM imidazole in 20 mM Tris (pH 8) buffer. Site directed mutagenesis was performed using Pfu DyeMix Forte DNA polymerase (Enzynomics, Daejeon, Korea). The mutation primers are listed in supplementary information (see Supplementary Table S2 online). The mutants were purified using the same protocol used for purification of the native proteins. ApeR was cloned into pET28a expression vector (Novagen, Madison, WI) using restriction enzymes *Nde*I and *Xho*I. The construct contains N-terminal 6-His tag, which is cleavable by thrombin. We expressed AbApeR using the same protocol as that used for AbApeO-AbApeC. We cleaved the 6-His tag after affinity chromatography and the protein was further purified using Q-Sepharose HP 5-ml column (GE Healthcare, Chicago, IL).

### Crystallization and structure determination

Crystallization of AbApeO-AbApeC and AbApeR was assessed using PEG/Ion HT screening kit (Hampton Research, USA) in a sitting drop plate. Needle crystals of AbApeO-AbApeC were grown in a buffer containing 0.2 M sodium malonate (pH 6.0) and 20% polyethylene glycol (PEG) 3350. AbApeR was crystallized in the crystallization buffer containing 0.2 M sodium phosphate dibasic (pH 9.2) and 20% PEG 3350. Crystals were transferred to the crystallization buffer containing the cryoprotectant 25% (v/v) ethylene glycol and flash cooled with liquid nitrogen for data collection at Pohang Accelerator Laboratory beamline 7A. For AbApeO-AbApeC, the diffraction dataset was processed at 1.88 Å using the program XDS (see Supplementary Table S1 online)^7^. The crystal structure was determined by molecular replacement with the coordinates of *X. doucetiae* ApeC/ApeO structure (PDB ID: 3U0F) using program Phaser implemented in Phenix program suite^25^. The structure of AbApeR diffracted to 1.85 and was determined by molecular replacement using the coordinates of *E. coli* KAS II structure (PDB ID: 1KAS). The structure was modeled using the program Coot and refined using the program Phenix^26,27^.

### *Spectrophotometric assay of *in vitro* APE production*

Recombinant proteins (holo ACP1, holo ACP2, AbApeH-AbApeP, AbApeQ, and AbFabD) were prepared as described previously^28^. Prior to the assay, 1 mM holo ACP1 was incubated with 1.25 mM 4-hydroxylbenzoic acid (4HBA), AbApeH-AbApeP, 3 mM ATP, and 10 mM MgCl_2_ for 1 h at room temperature (25 ºC). The product, 4HBA-ACP1 was used without any further purification. The reaction mixture (200 μl) comprised 11.7 mM malonyl-CoA, AbApeQ, AbFabD, AbApeH-AbApeP, 0.04 mM holo ACP2, and 0.075 mM 4HBA-ACP1. Reaction was initiated by adding 0.3 mM NADPH to the solution. Spectral scan was conducted every 30 s for the duration of 4 min using an Agilent 8453 UV–VIS spectrophotometer (Agilent, Santa Clara, CA).

### ITC

All ITC experiments were conducted using a MicroCal Auto-iTC200 (GE Healthcare, Chicago, IL) at the Korea Basic Science Institute (Ochang, Korea). Binding affinity was measured using 120 µl of 2.0 mM holo ACP1 or holo ACP2 and 370 µl of 0.1 mM AbApeO-AbApeC or AbApeR in 40 mM potassium phosphate buffer (pH 7.0). For each titration experiment, 3 µl of Tandem AbACPs was injected into KS-CLF for 4 s at intervals of 150 s at 25 °C. Overall, 19 injections were performed for each experiment and the data were analyzed using MicroCal Origin software. To prevent the dimerization of holo ACPs, 2 mM of 2-mercaptoethanol was added to the buffer prior to the experiments.

### Computational assessment of crystal structures

The structure of AbApeO-AbApeC was analyzed using the SiteMap tool in Maestro software package, 2020–2 release (Schrödinger, LLC, New York, NY). For the aryl polyene models in Fig. 7, carboxylate forms of 3EN and 6EN were docked into the active site by the application Glide from Maestro software package. Briefly, ligands were generated by LigPrep module using OPLS3e force field and receptor grid was set to include the whole cavity. Docking was conducted using standard precision mode to generate 10 poses, after which post-docking minimization was performed. For each docking procedure, the protein was kept as a rigid entity and the pose of the lowest energy was chosen for analysis.

## Supplementary Information

Supplementary Information.

## Data Availability

Authors declare that all the data that support the findings of this study are available from the corresponding author upon request. The three-dimensional structural coordinates have been deposited in the Protein Data Bank under accession numbers: 7F28, AbApeO-AbApeC; 7F27, AbApeR.
