# Supplementary material for: Structural basis of the complementary activity of two ketosynthases in aryl polyene biosynthesis
Source: Sci Rep. 2021 Aug 11;11:16340. doi: 10.1038/s41598-021-95890-y (PMC8358021; doi:10.1038/s41598-021-95890-y)
Supplement: Supplementary file 1 — Supplementary Information. [file 41598_2021_95890_MOESM1_ESM.pdf]

Supplementary information

Structural basis of the complementary activity of two ketosynthases in aryl polyene biosynthesis

Woo Cheol Lee, Sungjae Choi, Ahjin Jang, Jiwon Yeon, Eunha Hwang, and Yangmee Kim

Supplementary Table S1. Statistics of X-ray crystallographic data collection and refinement.

	**AbApeO-AbApeC**	**ApeR**
PDB ID	7F28	7F27
Resolution (Å)	39.05-1.88	40.68-1.80
Space group	$P2_1$	$P2_1$
Cell constants	a = 54.71, b = 104.24, c = 98.18 Å, β=98.2°	a = 62.58, b = 109.53, c = 122.90 Å and β = 97.4º.
R_{merge}	0.071 (0.44)	0.055 (0.45)
I/σI	12.76 (3.26)	13.64 (2.08)
Redundancy	3.68 (3.41)	3.58 (2.73)
Unique reflections	87522 (13251)	146463 (20604)
Completeness (%)	98.4 (92.5)	97.2 (84.8)
Wilson B-factor	21.8	27.2
R-factor	0.190 (0.334)	0.175 (0.337)
free-*R*	0.230 (0.365)	0.196 (0.319)
RMSD		
Bond lengths (Å)	0.007	0.007
Bond angles (°)	0.853	0.833
Overall *B*-factor	24.5	33.4
Ramachandran plot		
Favored (%)	97.1	97.4
Allowed (%)	2.64	2.48
Disallowed (%)	0.26	0.12

Note: Values in parentheses are of the outermost shells.

Supplementary Table S2. PCR primers used in this study. Restriction sites are underlined.

primer name	sequence
AbApeR_f	CG<u>CATATG</u>AAACGTGTAGTTGTGAC
AbApeR_r	CG<u>CTCGAG</u>TTATTGTTTAACACGTTTAAA
AbApeR_Y135A_f	TTAATGCAACCACTGCTATTCGCATGATGG
AbApeR_Y135A_r	CCATCATGCGAATAGCAGTGGTTGCATTAA

ApeC

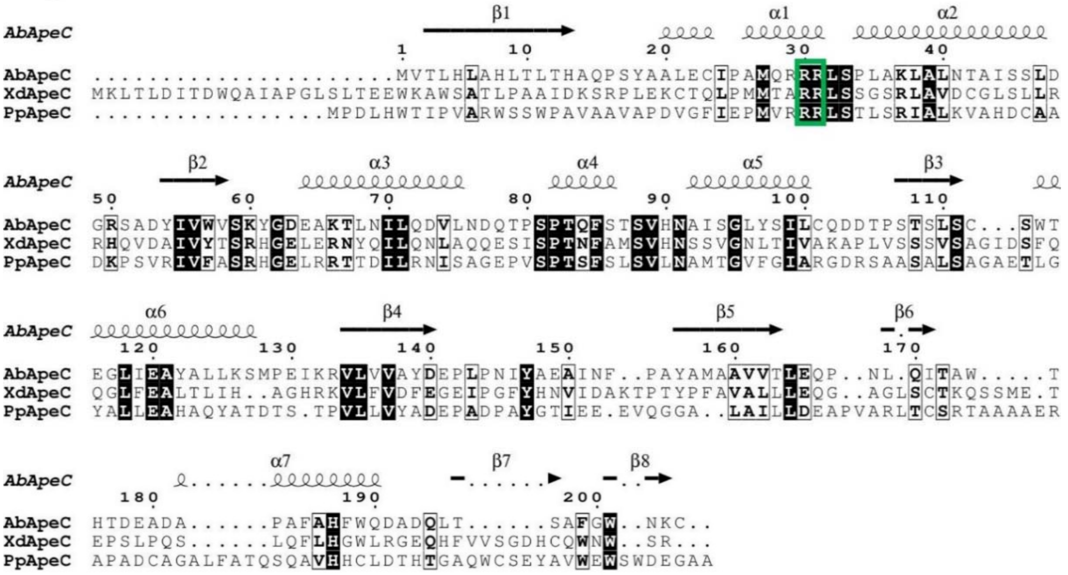

ApeO

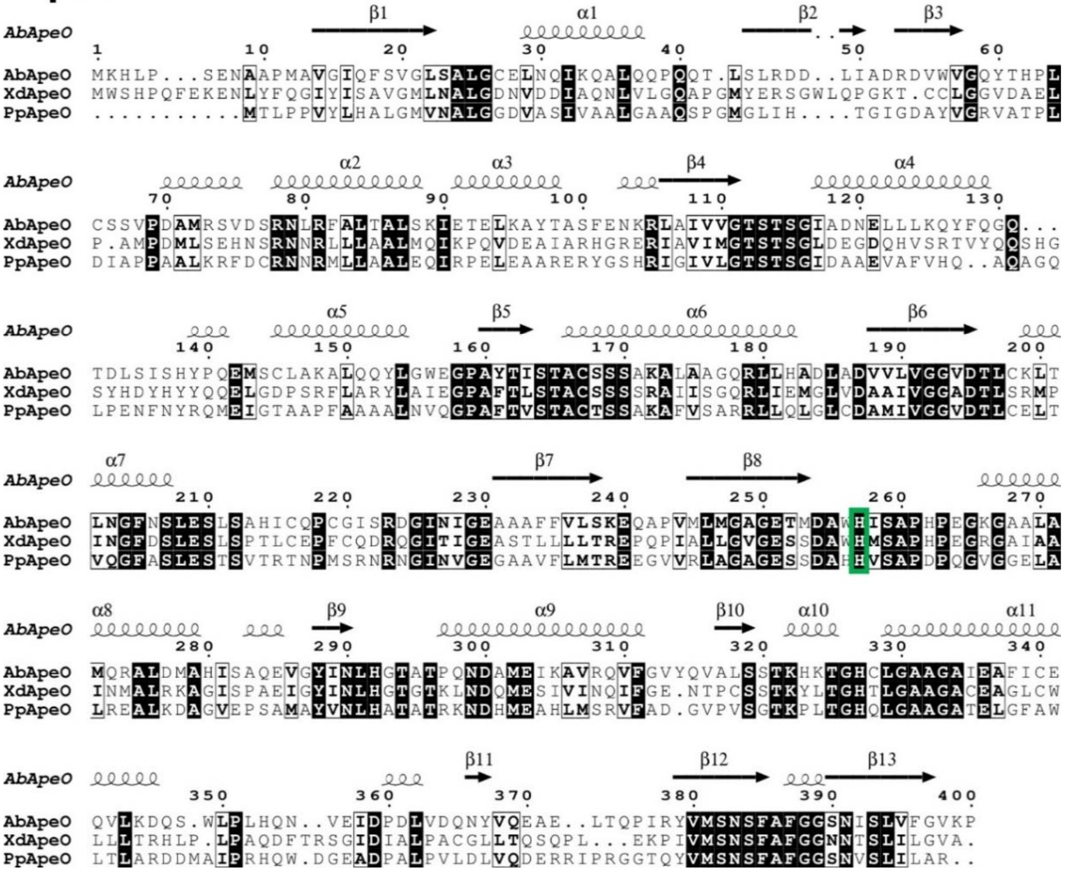

Supplementary Figure S1. Multiple sequence alignment of AbApeO-AbApeC.

Multiple sequence alignment of AbApeC or AbApeO with their homologs. Residues involved in ACP interaction are marked using green squares. XdApeC/XdApeO, *Xenoharbus doucetiae* ApeC/ApeO; PpApeC/PpApeO, *Paraburkholderia phymatum* STM815 ApeC/ApeO

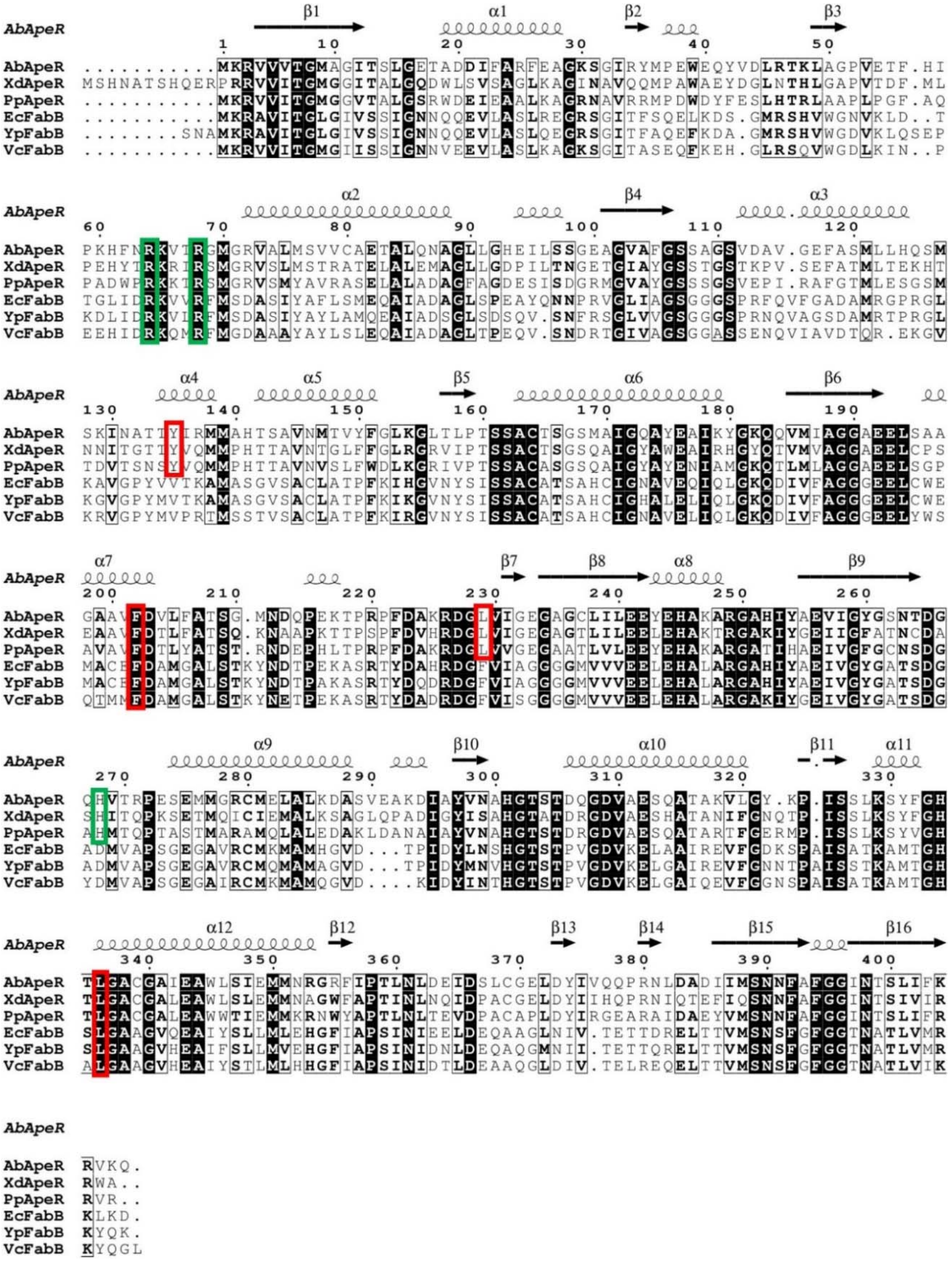

Supplementary Figure S2. Multiple sequence alignment of AbApeR and its homologs.

Multiple sequence alignment of AbApeR with homologs. Residues involved in ACP interaction and 4HB recognition are marked using green and red squares, respectively. EcFabB, *Escherichia coli* K12 FabB; YpFabB, *Yersinia pestis* FabB; VcFabB, *Vibrio cholerae* O1 biovar El Tor str. N16961 FabB.

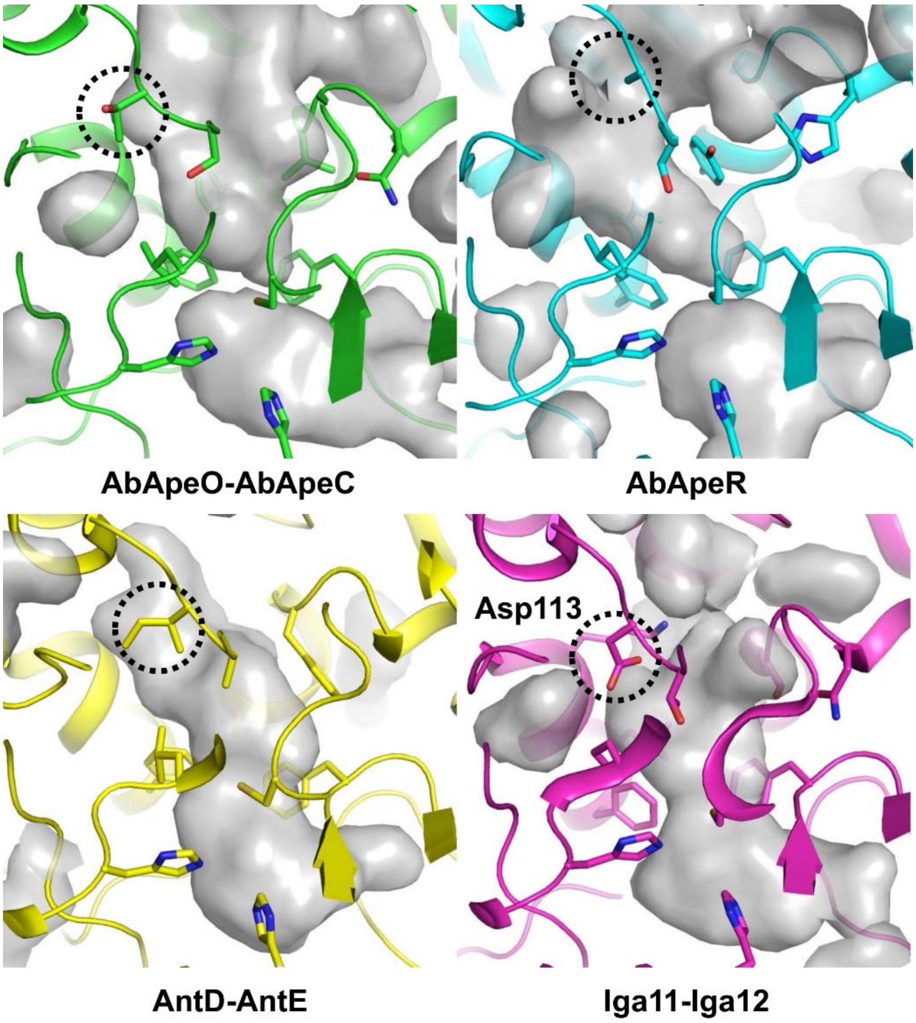

Supplementary Figure S3. Comparison of residues lining the cavity of various KS. The residues corresponding to Asp113 of Iga11-Iga12 are indicated using dashed circles.
